# Supplementary material for: Probing the surface charge of condensates using microelectrophoresis
Source: Nat Commun. 2024 Apr 26;15:3564. doi: 10.1038/s41467-024-47885-2 (PMC11053090; doi:10.1038/s41467-024-47885-2)
Supplement: Supplementary file 1 — Supplementary Information [file 41467_2024_47885_MOESM1_ESM.pdf]

Supplementary information

Probing the surface charge of condensates using microelectrophoresis

Merlijn H. I. van Haren[1], Brent S. Visser[1] and Evan Spruijt[1]*

[1] Institute for Molecules and Materials, Radboud University, Heyendaalseweg 135, 6523 AJ Nijmegen, The Netherlands. *Correspondence: e.spruijt@science.ru.nl

This PDF file includes:

Supplementary Note 1 to 6
Supplementary Figures 1 to 12
Supplementary Tables 1 to 3
Supplementary References

Other supplementary materials for this manuscript include the following:

Supplementary Movie 1

Supplementary Note 1: Electrophoretic mobility of droplets in electrolyte solutions

Particle electrophoresis and calculation of κR

The Smoluchowski equation (**E1**) is commonly used to describe electrophoresis of particles.[1]

$$v = \frac{\varepsilon_{\mathrm{r}}\varepsilon_0\zeta E}{\eta_{\mathrm{s}}} \qquad \textbf{(E1)}$$

Where v is the velocity of the particle, E the electric field strength, ε_{r} the dielectric constant, ε_0 the vacuum permittivity, ζ the zeta-potential, and η_{s} the viscosity of the medium. Equation **E1** is only valid in limits where the particle radius greatly exceeds size of the electrical double layer, defined by the Debye length (**E2**).[2] The Debye length (κ^{-1}) is defined as follows:

$$\kappa^{-1} = \sqrt{\frac{\varepsilon_{\mathrm{r}}\varepsilon_0 k_{\mathrm{B}}T}{2e^2 I}} \qquad \textbf{(E2)}$$

Where κ^{-1} is the Debye length, k_{B} the Boltzmann constant, T the temperature, e the elementary charge and I is the ionic strength in m^{-3}. Assuming a temperature of 298 K, a dielectric constant of 80, and expressing I in terms of molar concentration in all experiments, equation **E2** can be written as:

$$\kappa^{-1}(\mathrm{nm}) = \frac{1}{\sqrt{10.6\,C_{\mathrm{ion}}}} \qquad \textbf{(E3)}$$

Where C_{ion} is the electrolyte concentration in molar. For all coacervate systems considered in this research, we assume that C_{ion} is determined exclusively by the buffer, salts and counterions present in the dilute phase, while polyelectrolytes and proteins comprise the coacervate phase, from which we calculate C_{ion} with equation **E4**.[3]

$$C_{\mathrm{ion}} = \frac{1}{2}\sum_{i=1}^{N} c_i z_i^2 \qquad \textbf{(E4)}$$

Where c_i and z_i are the concentration and valence of ionic species i, respectively, and one half because we are including both cations and anions. For 50 mM Tris buffer pH 7.4, which contains 41 mM Tris-HCl and 9 mM Tris base, we have a C_{ion} of 41 mM. Together with the 5 mM Br^- and Na^+ counterions of K_n and D_n, respectively, gives a C_{ion} of 46 mM for each K_n/D_n coacervate sample. We validate the assumption of the similarity between dilute phases of different K_n/D_n samples by measuring the amount of current running through a channel slide filled with K_n/D_n coacervates with different lengths of polyelectrolyte (n), and confirm the relationship between current and ionic strength of the solution by calibration with NaCl solutions (**Supplementary Figure 1a**).[4] The experimentally measured current is the same within experimental uncertainty for all coacervate-containing samples, which confirms our assumption that the polyelectrolytes do not contribute significantly to the ionic strength.

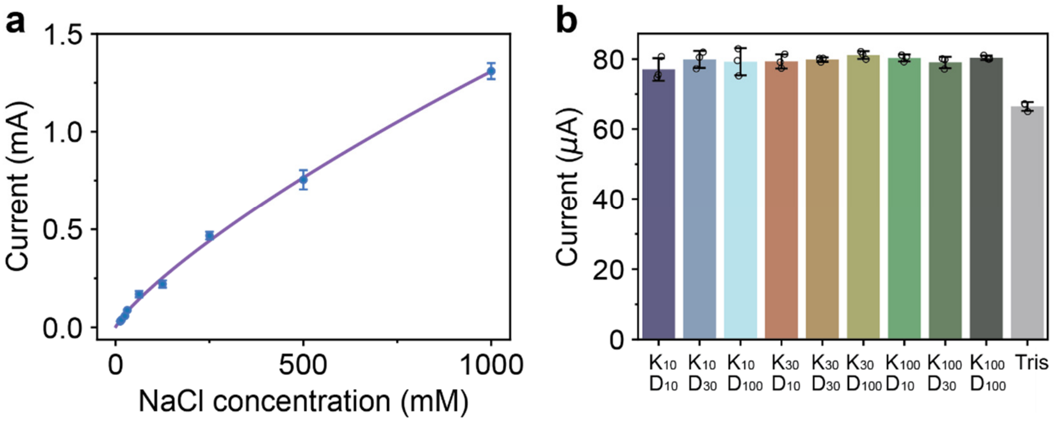

Supplementary Figure 1: Current measurements. **(a)** Current running through an Ibidi µ-channel slide containing different salt concentrations at an electric field strength of 1.9 V cm^{-1}. Error bars represent standard deviation (*n = 3*), data points are fitted with a power law *f(x) = a * x*^{b}. **(b)** Current running through a channel slide containing K_n/D_n with different lengths polyelectrolytes and 50 mM Tris buffer pH 7.4 at an electric field strength of 1.9 V cm^{-1}. Error bars represent standard deviation (*n = 3*) and raw values are displayed as dots.

For a calculated C_{ion} of 46 mM, we find that coacervates have a κ^{-1} of 1.4 nm (equation **E3**, **Supplementary Figure 2a**), which is significantly smaller than the typical coacervate radius of 1 to 10 µm (κR ~ 10^3 - 10^4), making these systems in theory valid for the Smoluchowski equation (**Supplementary Figure 2b**).

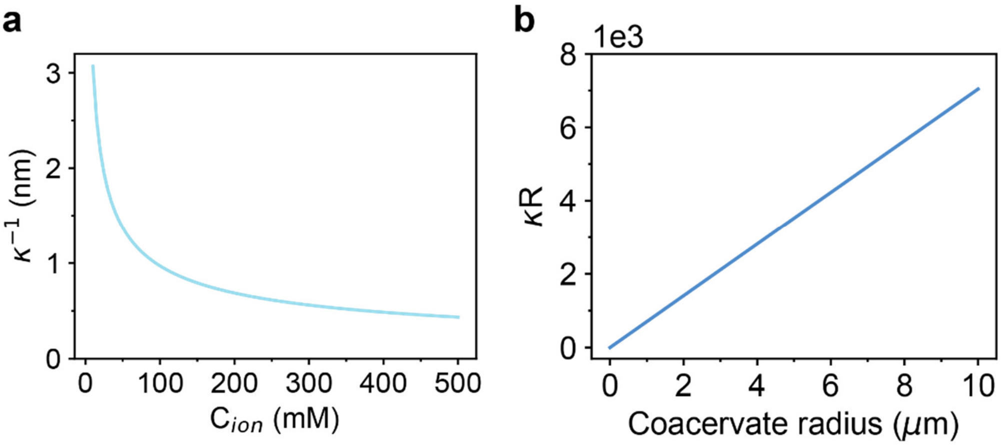

Supplementary Figure 2: Debye length and κR of coacervates. **(a)** Debye length of colloidal particles in water decays exponentially with increasing salt concentration, calculated with equation **E3**. **(b)** Coacervate radius over Debye length (κR) for 5 mM K_n/D_n coacervates in a 50 mM Tris buffer pH 7.4 (κ^{-1} = 1.43 nm). These calculations assume a dielectric constant of ε_r = 80, and temperature T = 298 K.

Calculation of zeta-potential of coacervates

To determine zeta-potential of coacervates, a theoretical framework by Ohshima *et al.* that describes the electrophoretic motion of mercury droplets in a potassium chloride solution was used.[6] The original theory assumes low Reynolds numbers of liquid flows outside and inside the droplets, an electric field magnitude that is weak enough that the velocity of particles is proportional to the field strength, and a spherical shape of the droplet during its motion. These assumptions are also valid for microelectrophoresis measurements on coacervate droplets. The only discrepancy is the assumption that electrolyte ions cannot penetrate the drop surface of mercury droplets, while coacervates are definitely penetrable by small ions. Because this property has a relatively small effect on the final velocity, we still considered the following equation to describe the electrokinetic movement of coacervate droplets:

$$\mu_e = \frac{3}{2}\left(\frac{e\zeta}{kT}\right)\left(\left(\frac{\eta_s}{3\eta_d + 2\eta_s}\right)\kappa R + \left(\frac{3\eta_d + \eta_s}{3\eta_d + 2\eta_s}\right) + 2e^{\kappa R}E_5(\kappa R) - \left(\frac{15\eta_d}{3\eta_d + 2\eta_s}\right)e^{\kappa R}E_7(\kappa R)\right) \quad \textbf{(E5)}$$

with a scaled electrophoretic mobility (μ_e) defined by:

$$\mu_e = \frac{3\eta_s e v}{2\varepsilon_r\varepsilon_0 k_B T E} \quad \textbf{(E6)}$$

and where E_n is the exponential integral of order n

$$E_n(x) = x^{n-1}\int_x^\infty \frac{e^{-t}}{t^n}dt \quad \textbf{(E7)}$$

For κR values larger than ~100, $e^{\kappa R}E_n(\kappa R)$ asymptotically approaches to $1/\kappa R$ (**Supplementary Figure 3**).

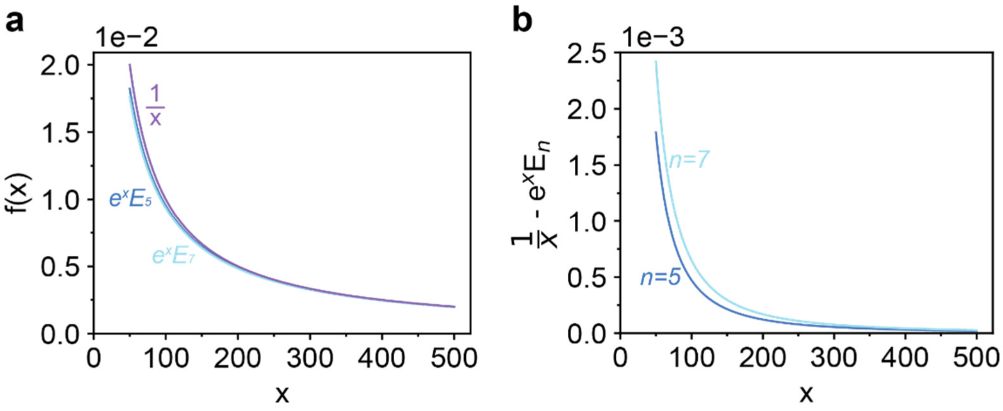

Supplementary Figure 3: The product of $e^{\kappa R}$ and equation **E7** asymptotes to $1/x$ (where $x = \kappa R$). **(a)** $f(x) = 1/x$ (purple) approaches $f(x) = e^xE_5$ (blue) and $f(x) = e^xE_7$ (light blue) for $x > 50$. **(b)** $1/x - e^xE_5$ (blue) and $1/x - e^xE_7$ (light blue) is zero for values of $x > 300$. For our systems, $x = \kappa R$, which is always higher than 500 for the coacervates studied here.

We therefore use this approximation for coacervates, as κR is always larger 1000 for coacervate droplets larger than 1 µm. This approximation allows us to simplify equation **E5** to **E8**.

$$\mu_e = \frac{3}{2}\left(\frac{e\zeta}{k_B T}\right)\left(\left(\frac{\eta_s}{3\eta_d + 2\eta_s}\right)\kappa R + \left(\frac{3\eta_d + \eta_s}{3\eta_d + 2\eta_s}\right) + \left(\frac{2}{\kappa R}\right) - \left(\frac{15\eta_d}{3\eta_d + 2\eta_s}\right)\left(\frac{1}{\kappa R}\right)\right) \quad \textbf{(E8)}$$

By further substituting **E6** into **E8**, we find the relation between droplet velocity v and all other system properties.

$$v = \frac{\varepsilon_r\varepsilon_0\zeta E}{\eta_s}\left(\left(\frac{\eta_s}{3\eta_d + 2\eta_s}\right)\kappa R + \left(\frac{3\eta_d + \eta_s}{3\eta_d + 2\eta_s}\right) + \left(\frac{2}{\kappa R}\right) - \left(\frac{15\eta_d}{3\eta_d + 2\eta_s}\right)\left(\frac{1}{\kappa R}\right)\right) \quad \textbf{(E9)}$$

Equation **E9** shows how droplet velocity scales linearly with the applied electric field strength and ζ potential of the droplet. However, it is not immediately clear how the velocity scales with increasing κR, as equation **E9** includes three separate terms containing κR. As can be seen in **Supplementary Figure 4**, droplet velocity scales linearly with κR when $\kappa R > 500$, assuming a coacervate viscosity of 100 mPa s.

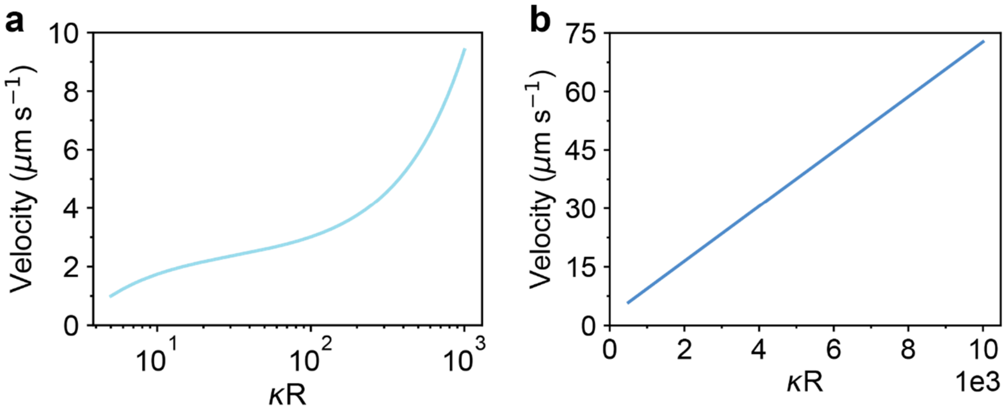

Supplementary Figure 4: Droplet velocity compared to *κR* values of coacervate droplets according to **E8**. **(a)** At small values of *κR*, not studied in this research, droplet velocity is inversely proportional to *κR*. **(b)** At *κR* > 500, valid for droplets with radius larger than 0.5 µm at physiological salt concentrations, velocity scales linearly with *κR*. These calculations assume a temperature of 298 K, ζ-potential of 10 mV, an applied electric field strength of 3 V cm^{-1}, a dilute phase viscosity of 0.89 mPa s and a droplet viscosity of 100 mPa s.

Equation **E9** can be simplified further by substituting η_s and η_d by the viscosity ratio η_r that represents the ratio of the coacervate phase viscosity compared to the viscosity of the surrounding solution.

$$\eta_r = \frac{\eta_d}{\eta_s} \qquad \textbf{(E10)}$$

Substituting **E10** into **E9** and further rewriting the equations gives **E11**.

$$v = \frac{\varepsilon_r \varepsilon_0 \zeta E}{\eta}\left(\left(\frac{1}{3\eta_r + 2}\right)\kappa R + \left(\frac{3\eta_r + 1}{3\eta_r + 2}\right) + \left(\frac{2}{\kappa R}\right) - \left(\frac{15\eta_r}{3\eta_r + 2}\right)\left(\frac{1}{\kappa R}\right)\right) \qquad \textbf{(E11)}$$

When taking into account that coacervate droplets typically have a viscosity that is significantly larger than their surrounding solution ($\eta_r >> 1$), and a radius that is larger than 1 µm (*κR* >> 100), **E11** can be further simplified to **E12** (equation 1 in main text), as the last three terms become negligible.

$$v = \frac{\varepsilon_r \varepsilon_0 \zeta E}{\eta_s}\left(1 + \frac{\kappa R}{3\eta_r}\right) \qquad \textbf{(E12)}$$

This equation looks very similar to the Smoluchowski equation (**E1**) but with and extra term that contains *κR* divided by the viscosity ratio η_r. **Equation 12** also shows the linear dependence on droplet radius, and its calculated values deviate no more than 1% when using *κR* of 500 to 10000 and a droplet viscosity η_d of 0.1 to 10 Pa s^{-1} ($\eta_r = 10^2$ to 10^4). The droplet viscosity η_d is highly variable for coacervates, with ranges from 0.1 to 10 Pa s.[7] At infinite viscosity, the radius dependent term in **E12** vanishes and particle movement can be described by the Smoluchowski equation (**Supplementary Figure 5**).

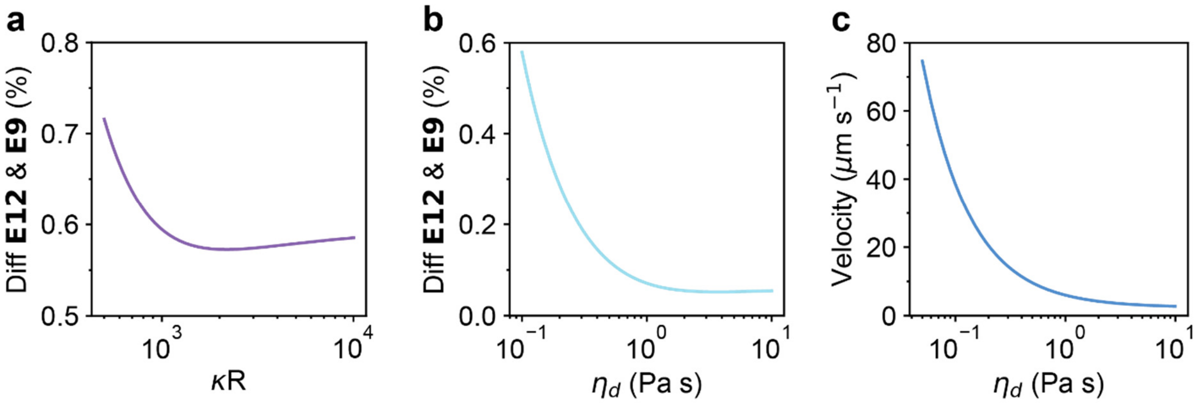

Supplementary Figure 5: Comparison between equation **E12** and **E9** and droplet velocity as a function of droplet viscosity according to equation **E12**. Difference between equation **E12** and **E9** depending on κR, with η_d = 100 mPa s **(a)** and depending on η_d with κR = 500 **(b)**. **(c)** Droplet velocity decays exponentially with increasing η_d (for κR = 5000). These calculations assume a temperature of 298 K, ζ-potential of 10 mV, an applied electric field strength of 3 V cm^{-1} and a dilute phase viscosity of 0.89 mPa s, which are all applicable to coacervates studied here.

To calculate the ζ-potential of coacervates, equation **E12** can be rewritten to **E13** (equation 2 in main text).

$$\zeta = \frac{3v\eta_{\mathrm{d}}}{\varepsilon_{\mathrm{r}}\varepsilon_0 E}\left(\frac{1}{3\eta_{\mathrm{r}} + \kappa R}\right) \qquad \textbf{(E13)}$$

Supplementary Note 2: Measurement of coacervate zeta potential by microelectrophoresis

To determine the zeta potential by microelectrophoresis, a microchannel containing coacervate solution was placed on an inverted microscope and incubated for 1 hour to allow coacervates to coalesce an settle on the glass modified glass surface (**Supplementary Figure 6**). The resulting coacervates have a typical have a radius of 2 to 10 µm and will move in two dimensions when an electric field is applied, which allows for single-droplet tracking by edge detection, without measuring the droplet mobility at different z-levels. After incubation, two silver electrodes connected to a direct-current power source are lowered in the opposing ends of the microchannel, and the solution is let to equilibrate for approximately 1 minute, as insertion of the electrodes can cause droplet movement because of a change in the water level. Normally, a potential of 5 V (3.13 V cm^{-1}, calculated for a distance of 1.6 cm between the electrodes) was applied to the coacervates and a time series of 100 frames during 10 seconds was recorded. If a 5 µm coacervate did not move faster than approximately 2 µm s^{-1}, the potential was increased by steps of 2 V until a desired response was observed. When the potential difference exceeded 10 V (6.25 V cm^{-1}), formation of gas bubbles by electrolysis could influence the timeseries by a sudden flow that sped up or slowed down droplets. When this occurred, the measurement was discarded and a new measurement was taken. For this reason, no measurements were conducted above a potential difference of 20 V (12.5 V cm^{-1}), as the frequency of gas bubbles disturbed almost every measurement. When 5 µm coacervates did not move faster than approximately 2 µm s^{-1}, a timeseries of 100 frames for 100 seconds instead of 10 seconds was recorded. It should be noted that the particle tracking script allows for a maximum coacervate displacement of one droplet radius per frame (max distance per frame = R), meaning a *N* µm coacervate can maximally move *10N* µm s^{-1} with a framerate of 10 fps, but only *N* µm s^{-1} for 1 fps. In this study, a framerate of 10 fps was always sufficient to capture all droplet movement under 6.25 V cm^{-1}, but care should be taken when lower framerates are used.

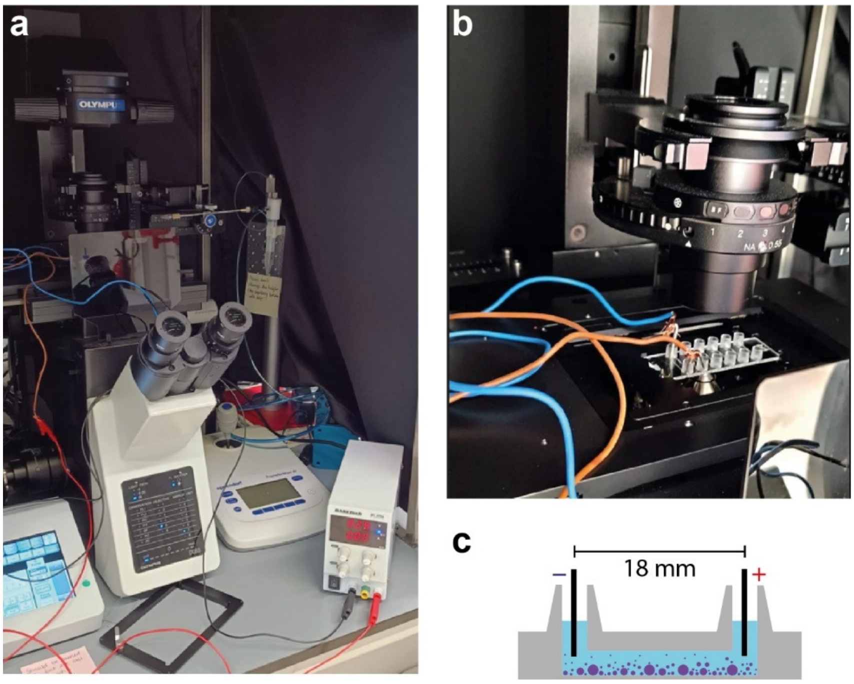

Supplementary Figure 6: Microelectrophoresis setup used in this study. **(a)** the cathode and anode are connected to a direct current power source by copper wires, the voltage between the two electrodes can be set at 0 to 30 V (0 to 18.75 V cm^{-1}). **(b)** Two silver electrodes with the cathode at the top and anode at the bottom inserted in a microchannel slide containing coacervate solution sit on an inverted microscope. **(c)** Schematic side view of the channel containing the electrodes and coacervates.

Normally, each sample was recorded at three different voltages. After the particle tracking algorithm was applied (**Script M1**), droplets that did not move, moved too much in the direction perpendicular to the applied field due to maneuvering around immobilized droplets, or droplets that suddenly slowed down or sped up due to surface passivation inhomogeneities were discarded (**Script M2**). This step also filtered out any mismatched droplets. According to equation **E12**, droplets bearing a nonzero surface charge should display electrophoretic mobility under any applied electric field. However, we typically observed that a (low) threshold electric field strength (E_0) was required for coacervates start moving. We interpret this as droplets weakly adhering to the modified glass slide. To compensate for this resistance, we calculate an effective electric field strength by subtracting an offset E_0 from the applied electric field E before calculating a coacervate ζ-potential (**Supplementary Figure 7**). The value of E_0 was later determined by starting the experiment at 0 V and gradually increasing the voltage until coacervates started moving. The resulting value for E_0 was not different for the two cases, but the last method was preferred as it reduced the total time of analysis.

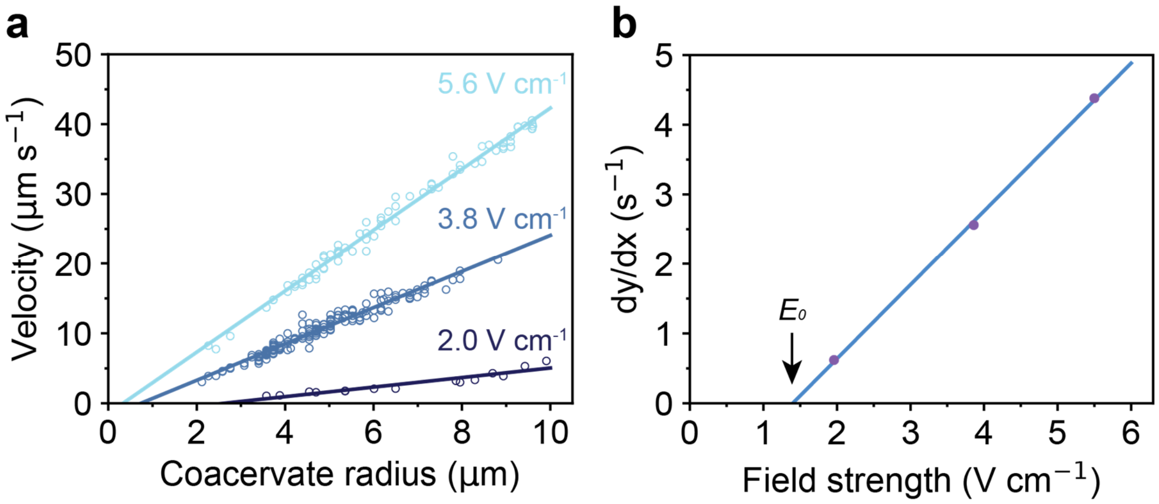

Supplementary Figure 7: Method for determining E_0 based on the velocity coefficient and applied electric field strength. **(a)** Motility of K_{10}/D_{10} coacervates at 2.0 V cm^{-1} (dark blue), 3.8 V cm^{-1} (blue) and 5.6 V cm^{-1} (light blue). **(b)** First derivative of **E12** as a function of droplet radius at different electric field strengths. E_0 is the intercept of the linear fit through the coefficients with the x-axis.

After accounting for E_0, the coacervate ζ-potential was calculated from the slope of the velocity as a function of radius (**Script M3**):

$$\frac{dv}{dR} = \frac{\varepsilon_\mathrm{r}\varepsilon_0\zeta E\kappa}{3\eta_\mathrm{d}} \qquad \textbf{(E14)}$$

$$\zeta = \frac{3\eta_\mathrm{d}}{\varepsilon_\mathrm{r}\varepsilon_0 E\kappa}\left(\frac{dv}{dR}\right) \qquad \textbf{(E15)}$$

To confirm that the friction of the condensate with the bottom surface of the sample due to a non-uniform contact angle of the coacervate in motion is minimal, we measured a z-stack of a typical coacervate droplet on in a passivated microchannel. Different side-on view show that the contact angle of the coacervate is very close to 180° (**Supplementary Figure 8**).

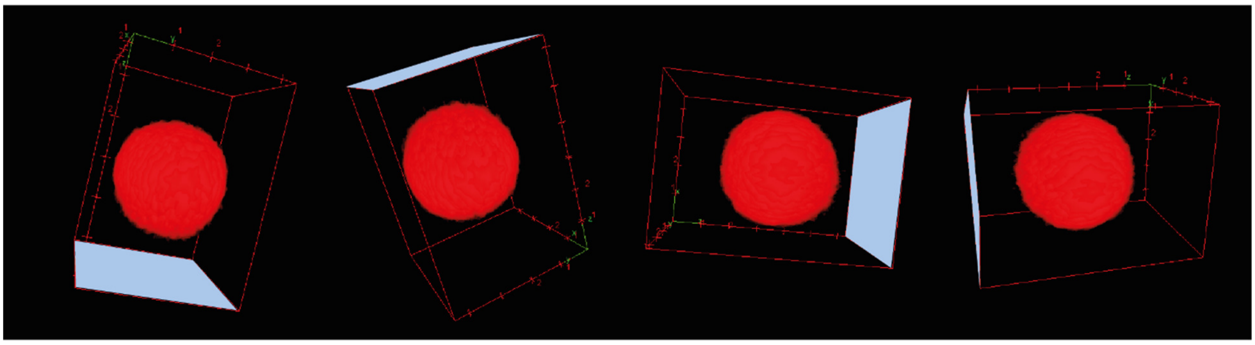

Supplementary Figure 8: Four different side-on views of a K_{10}/D_{10} coacervate droplet resting on a modified microchannel. The blue face indicates the direction of the bottom surface of the microchannel.

Supplementary Note 3: Raster image correlation spectroscopy and viscosity of coacervates

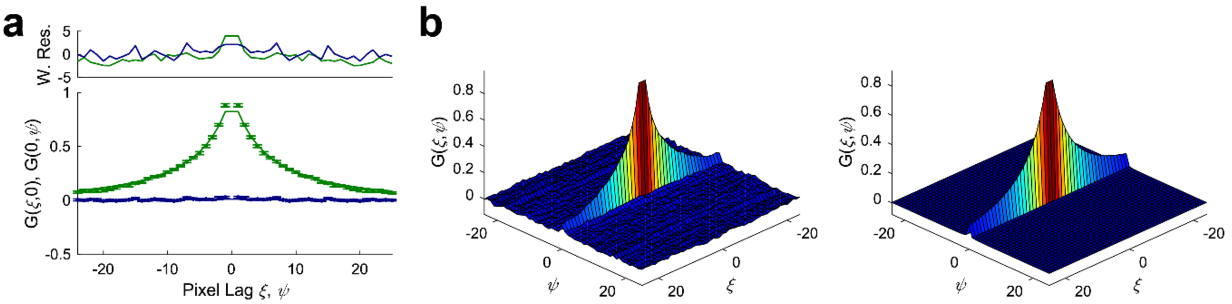

Supplementary Figure 9: Overview Raster Image Correlation Spectroscopy. Comparison of 2D autocorrelation function and its fit for K_{72}/ATP coacervates with TRITC-Dex(4.4k). **(a)** The ξ and ψ correlation (x and y) for each spatial pixel lag. Lines are the fit function, and scatter shows the data. **(b)** 2D ACF surface plot (left) and fit 2D ACF (right).

Supplementary Table 1: Fluorescent probes used in RICS for each coacervate system and its measured diffusion coefficient. Droplet viscosity was determined from the diffusion coefficient and label size by the Stokes-Einstein relation. Error is standard deviation (*n = 4* for all samples except for K_{72}/ATP (*n = 8*), NPM-1/polyU (*n = 2*) and protamine/NADH (*n = 2*).

Sample	Label used	Label size (nm)	*D* (µm^2 s^{-1})	η_d (mPa s)
K_{10}/D_{10}	TRITC-Dex (4.4k)	1.40e-9	7.4 ± 0.6	37 ± 4
K_{10}/D_{30}	TRITC-Dex (4.4k)	1.40e-9	2.6 ± 0.3	59 ± 6
K_{10}/D_{100}	TRITC-Dex (4.4k)	1.40e-9	1.8 ± 0.6	95 ± 33
K_{30}/D_{10}	TRITC-Dex (4.4k)	1.40e-9	1.1 ± 0.1	140 ± 9
K_{30}/D_{30}	TRITC-Dex (4.4k)	1.40e-9	1.2 ± 0.1	130 ± 14
K_{30}/D_{30} + 0.3 M NaCl	TRITC-Dex (4.4k)	1.40e-9	1.5 ± 0.1	110 ± 11
K_{30}/D_{100}	TRITC-Dex (4.4k)	1.40e-9	0.2 ± 0.0	690 ± 150
K_{100}/D_{10}	TRITC-Dex (4.4k)	1.40e-9	1.5 ± 0.2	110 ± 13
K_{100}/D_{30}	TRITC-Dex (4.4k)	1.40e-9	1.0 ± 0.1	160 ± 13
K_{100}/D_{100}	TRITC-Dex (4.4k)	1.40e-9	0.3 ± 0.2	560 ± 310
K_{100}/D_{100}	FITC-Dex (10k)	2.30e-9	0.2 ± 0.1	500 ± 300
K_{100}/D_{100} (2x [TRITC-Dex])	TRITC-Dex (4.4k)	1.40e-9	0.3 ± 0.1	590 ± 310
K_{72}/ATP	TRITC-Dex (4.4k)	1.40e-9	10 ± 1.1	15 ± 1.5
NPM-1/polyU	TRITC-Dex (4.4k)	1.40e-9	0.1 ± 0.0	1200 ± 170
RRP/polyU (0.75 M NaCl)	TRITC-Dex (4.4k)	1.40e-9	0.1 ± 0.0	1400 ± 220
Protamine/NADH	Alexa 488	6.13e-10	0.6 ± 0.2	621 ± 223
K_{10}/D_{10} + ATP (3 mM)	TRITC-Dex (4.4k)	1.40e-9	2.2 ± 0.5	75 ± 20

Supplementary Note 4: Electroosmotic flow in coacervate samples

Electroosmosis can occur when an electric field is applied to a capillary with charged walls. When an electric field is applied to the capillary containing an electrolyte solution, the excess counterions at the diffuse layer of the electrical double layer at the surface will drag the surrounding liquid along. Because the microchannel is orders of magnitude larger than the length scale of the electrical double layer, the electroosmotic flow can be considered as a plug flow throughout the whole channel.[8] To study the influence of this plug flow on the electrophoresis of coacervate droplets, we looked at the velocity of K_{10}/D_{10} droplets at a z-level 100 µm above the glass interface where electrophoresis is recorded. Because large coacervates settle to the glass surface quickly, only small droplets were recorded at this position (**Supplementary Figure 10a**). When analyzing their velocity, the droplets moved in the same direction, with a velocity similar to droplets at the glass interface (**Supplementary Figure 10b**). Because of this, we determined the electroosmotic flow to be negligible compared to the typical droplet velocities recorded during microelectrophoresis.

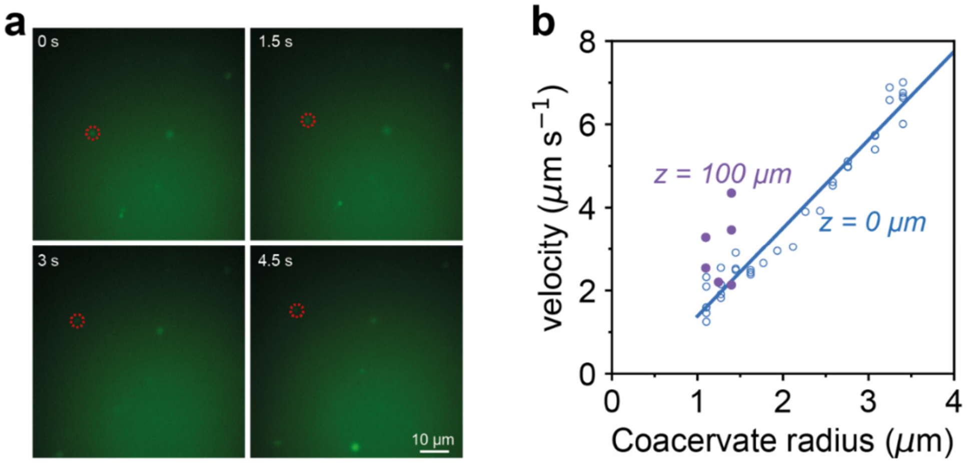

Supplementary Figure 10: Electroosmotic flow is negligible for coacervates during microelectrophoresis. **(a)** Movement of K_{10}/D_{10} coacervates containing 1 µM Alexa488 at a z-position of 100 µm above the glass slide. **(b)** Movement of K_{10}/D_{10} coacervates at the glass surface (blue) and at z = 100 µm (purple). The coacervates at the higher z position have the same directionality as at the glass surface, indicating the absence of an electroosmotic flow.

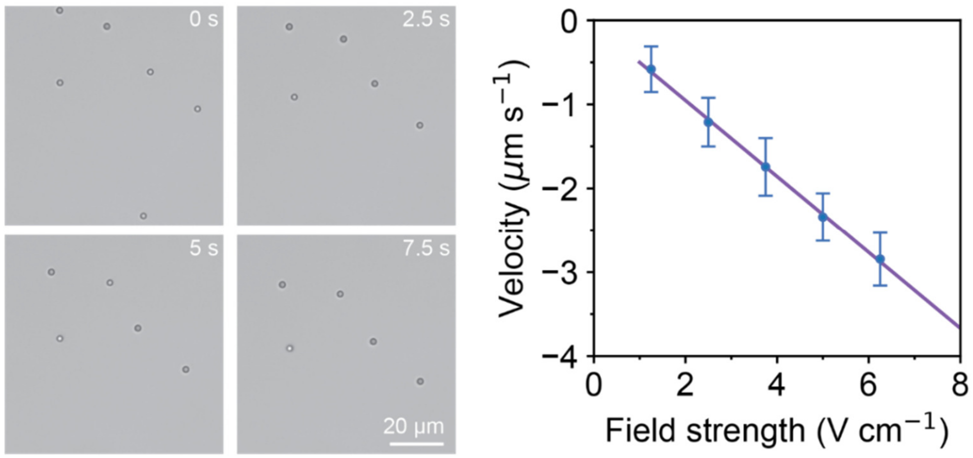

Supplementary Figure 11: Electrophoresis of negatively charged polystyrene beads. **(a)** The polystyrene beads move to the towards the anode, indicating that electroosmotic flow is negligible. **(b)** Bead velocity increases linearly with increasing field strength. A ζ-potential of -6.0 ± 0.1 mV (*n = 5*) was determined for the polystyrene beads using equation **E1**.

Supplementary Note 5: Zeta potential measurements by laser doppler electrophoresis

Our microelectrophoresis results were compared to laser doppler electrophoresis using a Malvern zetasizer, which is the standard method for determining coacervate ζ-potential. Laser doppler electrophoresis uses dynamic light scattering to detect particles. When an electric field is applied, usually in the range of 16-25 V cm^{-1}, the machine measures the electrophoretic mobility of the particles by a frequency shift of the light scattering signal, and converts this to a ζ-potential using the Smoluchowski equation **1**. As discussed in the text, this method does not take into account the radius-dependent electrophoretic mobility, the influence of Debye length (even at $\kappa R >> 1$) and droplet viscosity, which could lead to a wrong ζ-potential calculation. However, the sign of the ζ-potential calculated by either method should always correspond, as it purely dependent on whether the coacervates move towards the positive or negative electrode. Laser doppler electrophoresis was used to compare the ζ-potentials of K_n/D_n coacervates acquired from microelectrophoresis.

All samples were prepared with an identical composition to the K_n/D_n studied with microelectrophoresis. With microelectrophoresis, it was found that K_{10}/D_{10}, K_{30}/D_{10}, K_{30}/D_{30}, K_{100}/D_{10} and K_{100}/D_{30} had a positive surface charge, K_{10}/D_{100} had a negative surface charge and K_{10}/D_{30}, K_{30}/D_{100} and K_{100}/D_{100} were near neutral charge. The positively charged droplets found by microelectrophoresis are also detected as positive by laser doppler electrophoresis, as well as the negatively charged K_{10}/D_{100} coacervates. The K_{10}/D_{30}, K_{30}/D_{100} and K_{100}/D_{100} droplets that were near neutral charged are either negative or slightly positive when measured with laser doppler electrophoresis. It has to be noted that the ζ-potential distributions are extremely wide, and often cover both positive and negative values. This could be caused by caused by multiple factors that are inherent to coacervates measured by the zetasizer. The wide distributions are caused by the radius-dependent electrophoretic mobility, as larger coacervates will move faster in the electric field. Additionally, the zetasizer applies electric fields with strengths exceeding 10 V cm^{-1}, which leads to vacuole formation in coacervates and could greatly influence their electrophoretic mobility. Coacervates could also move in the vertical direction by sedimentation, which would introduce extra movement in the sample that could be detected as electrophoretic mobility. Overall the signs of the ζ-potentials of K_n/D_n coacervates are reasonably between microelectrophoresis and laser doppler electrophoresis, which suggests that we are not measuring the electroosmotic flow of the solution caused by electrophoresis.

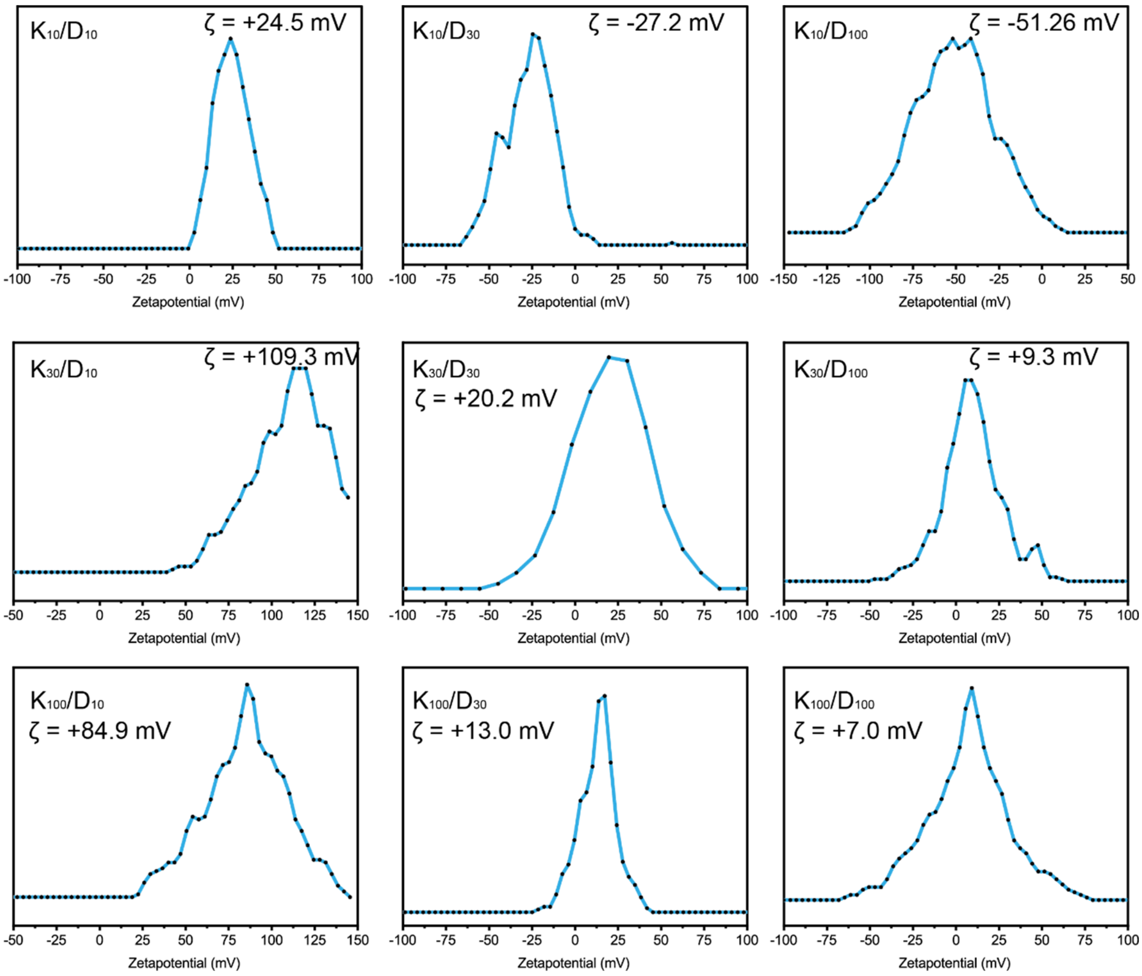

Supplementary Figure 12: ζ-potentials of K_n/D_n coacervates determined by laser doppler electrophoresis and electrophoretic light scattering. Coacervates consist of 5 mM K_n and D_n (monomer concentrations) in 50 mM Tris pH 7.4 buffer.

Supplementary Note 6: Zeta potential calculation parameters and statistical parameters

Supplementary Table 2: Parameters used to calculate ζ-potentials from microelectrophoresis. C_{ion} was calculated from the buffer concentration and added sodium chloride. η_d was determined by RICS. E_0 was determined by testing the lowest electric field strength at which droplets moved. When η_d is noted with a *, the viscosity was assumed to be similar to other Coacervates (concentration ratio's K_{10}/D_{30}) or was linearly interpolated (1 and 2 mM ATP K_{10}/D_{10}). Other parameters used to calculate the ζ-potential with **E15** are T = 298 K and η_s = 0.89 mPa s. Percentage of moving droplets is calculated from all droplet traces, and droplets are counted as moving when their velocity was faster than 0.5 µm s^{-1}. Samples denoted with a * have a near-zero ζ-potential, which means their electrophoretic mobility is also near zero, hence the low percentage.

**Sample**	**C_{ion} (mM)**	**η_d (mPa s)**	***E* (V)**	**E_0 (V)**	**ζ (mV)**	**Moving drops**
K_{10}/D_{10}	46	37 ± 4	6.17	2.4	+2.5 ± 0.1	98.4%
K_{10}/D_{30}	46	59 ± 6	6.17	1.0	-0.1 ± 0.0	55.9%*
K_{10}/D_{30} (2:1)	48.5	59 ± 6*	6.00	1.5	+8.6 ± 1.3	100%
K_{10}/D_{30} (1:2)	48.5	59 ± 6*	6.00	1.0	-4.4 ± 0.2	100%
K_{10}/D_{100}	46	95 ± 33	6.17	2.9	-3.1 ± 0.5	80.8%
K_{30}/D_{10}	46	140 ± 9	6.17	2.4	+17 ± 1.3	99.1%
K_{30}/D_{30}	46	130 ± 14	6.17	1.0	+12 ± 1.2	98.8%
K_{30}/D_{30} (0.3 M NaCl)	346	110 ± 11	3.14	1.5	+8.3 ± 0.5	99.1%
K_{30}/D_{100}	46	690 ± 150	6.17	1.0	-2.9 ± 0.3	15.8%*
K_{100}/D_{10}	46	110 ± 13	6.14	0.7	+8.5 ± 1.3	97.8%
K_{100}/D_{30}	46	160 ± 13	6.14	0.8	+8.5 ± 1.1	87.3%
K_{100}/D_{100}	46	560 ± 310	6.14	1.0	-0.6 ± 0.1	1.2%*
K_{72}/ATP	30.8	15 ± 1.5	6.00	2.0	+0.1 ± 0.0	97.2%
RRP/polyU (0.75 M NaCl)	797.2	1400 ± 220	6.00	1.5	-2.7 ± 0.6	92.7%
NPM1/polyU	267.6	1200 ± 170	6.00	2.1	-11 ± 1.4	80.3%
Protamine/NADH	49.6	620 ± 220	7.00	0.5	+1.9 ± 0.3	99.5%
K_{10}/D_{10} + ATP (1 mM)	47	50*	6.14	1.3	+0.0 +- 0.2	13.5%*
K_{10}/D_{10} + ATP (2 mM)	48.5	62*	6.14	1.4	-0.7 +- 0.2	95.2%
K_{10}/D_{10} + ATP (3 mM)	50	75 ± 20	6.14	2.4	-1.1 +- 0.3	96.7%

Supplementary Table 3: Statistics reporting summary. *n* represents the number of individual droplets measured to acquire the presented result. Two equations were used to fit the data. A linear equation (*mx + b*) or a Gaussian probability density function, accompanied with a cofactor (*C*) so raw data can presented without normalization (*C/(σ*(2pi)^0.5*exp(-0.5((x-μ)/σ)^2)*). For the linear fits an R^2 is given, while for the probability density function the mean (*μ*) and standard deviation (*σ*) are given

Main text Figure	*n*	*Fit*	R^2	*μ* (mV)	*σ (mV)*
1d	160	2.6x – 1.9	0.956		
1e purple	160	$16.7 / (\sigma * (2\pi)^{0.5}) * e^{-0.5((x-\mu)(x-\mu)/(\sigma * \sigma))}$		2.5	0.1
1e blue	160	$1298 / (\sigma * (2\pi)^{0.5}) * e^{-0.5((x-\mu)(x-\mu)/(\sigma * \sigma))}$		45	17
1f	160	$16.7 / (\sigma * (2\pi)^{0.5}) * e^{-0.5((x-\mu)(x-\mu)/(\sigma * \sigma))}$		2.5	0.1
1g purple	314	3.6x + 1.2	0.947		
1g blue	369	3.5c + 0.1	0.969		
1h purple	314	$94.9 / (\sigma * (2\pi)^{0.5}) * e^{-0.5((x-\mu)(x-\mu)/(\sigma * \sigma))}$		8.4	0.4
1h blue	282	$194 / (\sigma * (2\pi)^{0.5}) * e^{-0.5((x-\mu)(x-\mu)/(\sigma * \sigma))}$		12	0.5
2a K_{10}/D_{10}	160	2.6x – 1.9	0.956		
2a K_{10}/D_{30}	35	-0.2x – 0.0	0.722		
2a K_{10}/D_{100}	71	-1.1x + 0.9	0.810		
2a K_{30}/D_{10}	94	6.2x+11	0.858		
2a K_{30}/D_{30}	141	5.0x+11	0.838		
2a K_{30}/D_{100}	38	-0.2x+1.9	0.809		
2a K_{100}/D_{10}	61	4.6x + 7.9	0.880		
2a K_{100}/D_{30}	98	3.0x + 4.6	0.810		
2a K_{100}/D_{100}	187	0.1x + 0.4	0.504		
2b K_{10}/D_{10}	160			2.5	0.1
2b K_{10}/D_{30}	35			-0.1	0.0
2b K_{10}/D_{100}	71			-3.1	0.5
2b K_{30}/D_{10}	94			17	1.3
2b K_{30}/D_{30}	141			12	1.2
2b K_{30}/D_{100}	38			-2.9	0.3
2b K_{100}/D_{10}	61			8.5	1.3
2b K_{100}/D_{30}	98			8.5	1.1
2b K_{100}/D_{100}	187			-0.6	0.1
2d red	95	7.0x + 3.3	0.830		
2d grey	139	0.0x + 0.1	0.025		
2d blue	82	-4.0x – 3.0	0.954		
3a Linear	500	0.1x + 0.7	0.740		
3a Gauss	500	$2.9 / (\sigma * (2\pi)^{0.5}) * e^{-0.5((x-\mu)(x-\mu)/(\sigma * \sigma))}$		0.05	0.01
3b 0.00 M NaCl	112	0.0x + 0.0	0.038		
3b 0.25 M NaCl	123	-0.0x + 0.2	0.087		
3b 0.50 M NaCl	55	-0.1x – 1.0	0.497		
3b 0.75 M NaCl	78	-0.4x – 1.9	0.785		
3b Gauss	78	$32.1 / (\sigma * (2\pi)^{0.5}) * e^{-0.5((x-\mu)(x-\mu)/(\sigma * \sigma))}$		-2.8	0.48
3c Linear	103	-0.9x – 4.8	0.604		
3c Gauss	103	$80.6 / (\sigma * (2\pi)^{0.5}) * e^{-0.5((x-\mu)(x-\mu)/(\sigma * \sigma))}$		-11	1.19
3d Linear	140	0.2x + 2.0	0.821		
3d Gauss	140	$19.2 / (\sigma * (2\pi)^{0.5}) * e^{-0.5((x-\mu)(x-\mu)/(\sigma * \sigma))}$		1.9	0.15
4a K_{10}/D_{10}	160	2.6x – 1.9	0.956		
4a + 1 mM ATP	44	0.0x + 0.0	0.025		
4a + 2 mM ATP	171	-0.7x – 1.1	0.506		
4a + 3 mM ATP	196	-1.1x – 1.2	0.619		
4c pLys/pGlu	101	2.6x + 0.7	0.900		
4c α-syn	238	-0.0x + 0.4	0.016		
4c ATP	29	0.3x + 6.9	0.436		

Supplementary References

1. Smoluchowski M. Contribution to the theory of electro-osmosis and related phenomena. *Bull Int Acad Sci Cracovie* **184**, (1903).
2. Berg JC. *An introduction to interfaces & colloids: the bridge to nanoscience*. World Scientific (2010).
3. Solomon T. The definition and unit of ionic strength. *Journal of Chemical Education* **78**, 1691 (2001).
4. Rao SM, Thyagaraj T. Role of direction of salt migration on the swelling behaviour of compacted clays. *Applied clay science* **38**, 113-129 (2007).
5. Dukhin A, Parlia S, Somasundaran P. Ion-pair conductivity theory V: critical ion size and range of Ion-Pair existence. *Journal of The Electrochemical Society* **165**, E784 (2018).
6. Ohshima H, Healy TW, White LR. Electrokinetic phenomena in a dilute suspension of charged mercury drops. *Journal of the Chemical Society, Faraday Transactions 2: Molecular and Chemical Physics* **80**, 1643-1667 (1984).
7. Yewdall NA, André AA, Lu T, Spruijt E. Coacervates as models of membraneless organelles. *Current Opinion in Colloid & Interface Science* **52**, 101416 (2021).
8. Rashidi M, Zargartalebi M, Benneker AM. Mechanistic studies of droplet electrophoresis: A review. *Electrophoresis* **42**, 869-880 (2021).
