# Supplementary material for: Probing the surface charge of condensates using microelectrophoresis
Source: Nat Commun. 2024 Apr 26;15:3564. doi: 10.1038/s41467-024-47885-2 (PMC11053090; doi:10.1038/s41467-024-47885-2)
Supplement: Supplementary file 2 — Reporting Summary [file 41467_2024_47885_MOESM2_ESM.pdf]

nature portfolio

Corresponding author(s): Spruijt

Last updated by author(s): Apr 9, 2024

Reporting Summary

Nature Portfolio wishes to improve the reproducibility of the work that we publish. This form provides structure for consistency and transparency in reporting. For further information on Nature Portfolio policies, see our Editorial Policies and the Editorial Policy Checklist.

Statistics

For all statistical analyses, confirm that the following items are present in the figure legend, table legend, main text, or Methods section.

n/a	Confirmed	
[ ]	[x]	The exact sample size (*n*) for each experimental group/condition, given as a discrete number and unit of measurement
[ ]	[x]	A statement on whether measurements were taken from distinct samples or whether the same sample was measured repeatedly
[x]	[ ]	The statistical test(s) used AND whether they are one- or two-sided<br>*Only common tests should be described solely by name; describe more complex techniques in the Methods section.*
[x]	[ ]	A description of all covariates tested
[x]	[ ]	A description of any assumptions or corrections, such as tests of normality and adjustment for multiple comparisons
[ ]	[x]	A full description of the statistical parameters including central tendency (e.g. means) or other basic estimates (e.g. regression coefficient) AND variation (e.g. standard deviation) or associated estimates of uncertainty (e.g. confidence intervals)
[x]	[ ]	For null hypothesis testing, the test statistic (e.g. *F*, *t*, *r*) with confidence intervals, effect sizes, degrees of freedom and *P* value noted<br>*Give P values as exact values whenever suitable.*
[x]	[ ]	For Bayesian analysis, information on the choice of priors and Markov chain Monte Carlo settings
[x]	[ ]	For hierarchical and complex designs, identification of the appropriate level for tests and full reporting of outcomes
[x]	[ ]	Estimates of effect sizes (e.g. Cohen's *d*, Pearson's *r*), indicating how they were calculated

Our web collection on statistics for biologists contains articles on many of the points above.

Software and code

Policy information about availability of computer code

Data collection	No custom code was used for data collection
Data analysis	Code used for data analysis is deposited in the Radboud Data Repository together with the source data and is available under accession code https://doi.org/10.34973/yrs1-et97.

For manuscripts utilizing custom algorithms or software that are central to the research but not yet described in published literature, software must be made available to editors and reviewers. We strongly encourage code deposition in a community repository (e.g. GitHub). See the Nature Portfolio guidelines for submitting code & software for further information.

Data

Policy information about availability of data

All manuscripts must include a data availability statement. This statement should provide the following information, where applicable:

- Accession codes, unique identifiers, or web links for publicly available datasets
- A description of any restrictions on data availability
- For clinical datasets or third party data, please ensure that the statement adheres to our policy

All data supporting the findings of this study are available within the article, in the Supplementary information and supplementary data files. Source data are deposited in the Radboud Data Repository (https://data.ru.nl), and are available under accession code https://doi.org/10.34973/yrs1-et97.

Research involving human participants, their data, or biological material

Policy information about studies with human participants or human data. See also policy information about sex, gender (identity/presentation), and sexual orientation and race, ethnicity and racism.

Reporting on sex and gender	N/A
Reporting on race, ethnicity, or other socially relevant groupings	N/A
Population characteristics	N/A
Recruitment	N/A
Ethics oversight	N/A

Note that full information on the approval of the study protocol must also be provided in the manuscript.

Field-specific reporting

Please select the one below that is the best fit for your research. If you are not sure, read the appropriate sections before making your selection.

- [x] Life sciences
- [ ] Behavioural & social sciences
- [ ] Ecological, evolutionary & environmental sciences

For a reference copy of the document with all sections, see nature.com/documents/nr-reporting-summary-flat.pdf

Life sciences study design

All studies must disclose on these points even when the disclosure is negative.

Sample size	All experiments were carried out in solution or dispersion and volumes used were determined by the microscopic flow chamber. The concentrations were based on results with other coacervate systems formed by molecules of similar size reported by us and other in literature. These sample sizes were sufficient as more than 100 droplets were typically visible in the field of view of the microscope. Data points for all droplets analyzed are included in all figures. The number of droplets analyzed and the fraction of moving droplets is reported for each experiment in Supplementary information, Table S2. Parameters and n used for fits are present in Table S3.
Data exclusions	Objective criteria for excluding droplets from the analysis (droplets that did not move, or suddenly slowed down/accelerated due to surface inhomogeneities) are described in the methods section.
Replication	Experiments for certain coacervate compositions were performed in triplicate to confirm our method was consistent. For every experiment the data points of all droplets analyzed are included in the figures.
Randomization	This is not relevant to our study. We prepared and analyzed in vitro samples of peptides to determine their surface charge. We do not compare separate groups of samples/organisms/participants against each other using statistical tests.
Blinding	This is not relevant to our study. We prepare samples containing coacervates ourselves and need to know exactly what each sample contains in order to plot the surface charge properly.

Reporting for specific materials, systems and methods

We require information from authors about some types of materials, experimental systems and methods used in many studies. Here, indicate whether each material, system or method listed is relevant to your study. If you are not sure if a list item applies to your research, read the appropriate section before selecting a response.

Materials & experimental systems

n/a	Involved in the study	
x		Antibodies
x		Eukaryotic cell lines
x		Palaeontology and archaeology
x		Animals and other organisms
x		Clinical data
x		Dual use research of concern
x		Plants

Methods

n/a	Involved in the study	
x		ChIP-seq
x		Flow cytometry
x		MRI-based neuroimaging

Plants

Seed stocks	N/A
Novel plant genotypes	N/A
Authentication	N/A
