# Supplementary material for: Probing the surface charge of condensates using microelectrophoresis
Source: Nat Commun. 2024 Apr 26;15:3564. doi: 10.1038/s41467-024-47885-2 (PMC11053090; doi:10.1038/s41467-024-47885-2)
Supplement: Supplementary file 4 — Description of Additional Supplementary Files [file 41467_2024_47885_MOESM4_ESM.pdf]

File name: Supplementary Movie 1

Description: K_{10}/D_{10} coacervates in an electric field of 3.84 V cm^{-1}. The droplets move in the direction of the cathode, indicating that they are positively charged. Big droplets move faster than small droplets and their motion can be described by equation 1 in the main text. Droplet traces, velocities and ζ-potentials obtained from these droplets can be found in Figure 1c-f of the main text.
